# Prognostic factors and overall survival in pelvic Ewing's sarcoma and chordoma: A comparative SEER database analysis

**DOI:** 10.1016/j.heliyon.2024.e37013

**Published:** 2024-08-28

**Authors:** Wanyun Tang, Runzhuo Li, Xiaoying Lai, Xiaohan Yu, Renjian He

**Affiliations:** a Department of Orthopedics, Zigong First People's Hospital, Zigong, China; b Department of Digestion,The First People's Hospital of Yibin, Yibin, China; c Department of General Surgery, Dandong Central Hospital, China Medical University, Dandong, China

**Keywords:** Pelvic Ewing's sarcoma, Pelvic chordoma, Prediction model, Nomogram, SEER database

## Abstract

**Background:**

This study aimed to develop and validate nomograms to predict overall survival (OS) for pelvic Ewing's sarcoma (EWS) and chordoma, identify prognostic factors, and compare outcomes between the two conditions.

**Methods:**

We identified patients diagnosed with pelvic EWS or chordoma from the SEER database (2001–2019). Independent risk factors were identified using univariate and multivariate Cox regression analyses, and these factors were used to construct nomograms predicting 3-, 5-, and 10-year OS. Validation methods included AUC, calibration plots, C-index, and decision curve analysis (DCA). Kaplan-Meier curves and log-rank tests compared survival differences between low- and high-risk groups.

**Results:**

The study included 1175 patients (EWS: 611, chordoma: 564). Both groups were randomly divided into training (70 %) and validation (30 %) cohorts. OS was significantly higher for chordoma. Multivariate analysis showed year of diagnosis, income, stage, and surgery were significant for EWS survival, while age, time to treatment, stage, and surgery were significant for chordoma survival. Validation showed the nomograms had strong predictive performance and clinical utility.

**Conclusions:**

The nomograms reliably predict overall survival (OS) in pelvic EWS and chordoma, helping to identify high-risk patients early and guide preventive measures. The study also found that survival rates are significantly higher for chordoma, highlighting different prognostic profiles between EWS and chordoma.

## 1 Introduction

Ewing's sarcoma (EWS) and chordoma are rare primary malignant bone tumors [1,2]. EWS has an estimated incidence of 2.9 per million per year, occurring predominantly in children and adolescents, while chordoma occurs in 0.08 per 100,000, typically in older adults [3,4]. Both demonstrate a predilection for the axial skeleton and pelvic localization [5,6]. With multimodal therapy incorporating chemotherapy, radiation, and surgery, the 5-year overall survival (OS) rate for non-metastatic EWS now exceeds 70 %, but remains only 30–40 % when metastases are present at diagnosis [7]. Chordomas have a relatively better prognosis, with reported 5-year OS ranging from 80 to 90 % % for sacral tumors and only 30–50 % for recurrent disease [8].

Several prognostic factors have been reported to significantly influence clinical outcomes in EWS and chordoma independently, including age, tumor size, surgical resection extent, radiation response, and histological grade [7,9]. However, due to the rarity of these malignancies, most studies were small, retrospective case series examining.

Pelvic EWS and chordoma share several similarities, including anatomical location, locally aggressive behavior, and challenging surgical margins [[10], [11], [12], [13]]. However, they differ in patient demographics, metastatic potential, and sensitivity to chemotherapy/radiation. Understanding how prognostic factors and survival may differ between pelvic EWS and chordoma can better guide tailored treatment decision making for these rare tumors.

In this study, we utilize the Surveillance, Epidemiology, and End Results (SEER) database to conduct a comparative analysis of prognostic factors and overall survival between pelvic EWS and chordoma. Findings aim to delineate clinical and demographic differences between these rare pelvic malignancies to inform management.

## 2 Background

EWS is a highly aggressive tumor primarily affecting children and young adults, characterized by its origin in bone or soft tissue [14]. It is associated with specific chromosomal translocations, most commonly t(11; 22)(q24; q12), which results in the EWSR1-FLI1 fusion gene [15]. Chordoma, on the other hand, is a slow-growing, malignant bone tumor thought to arise from remnants of the notochord, with a predilection for the axial skeleton, including the sacrococcygeal region [16].

The pelvis is a common site for both EWS and chordoma, contributing to significant morbidity and challenging therapeutic management due to the complex anatomy and the need for extensive surgical resection [17,18]. Prognosis for patients with pelvic EWS and chordoma varies widely, influenced by factors such as age, tumor size, metastasis at diagnosis, and treatment modalities including surgery, chemotherapy, and radiotherapy [[19], [20], [21], [22], [23], [24]].

Despite advancements in treatment, the prognosis for EWS remains poor, with a 5-year survival rate of approximately 65 % in localized cases and significantly lower in metastatic disease [25]. Chordoma, although generally slower growing, also presents a therapeutic challenge due to its high recurrence rate and resistance to conventional chemotherapy, with a 5-year survival rate ranging from 50 % to 80 %, depending on the location and extent of the disease [19,[26], [27], [28], [29]].

## 3 Literature review

We also summarize many recent similar studies in Supplementary Table 1. For EWS, the study by Mathew et al. [30] focused on EWS in non-skeletal locations with a sample size of 47 cases, finding that tumor location and positive surgical margins significantly impacted prognosis. Dimosthenis Andreou et al. [21] studied 1411 cases of pelvic EWS, identifying tumor location, treatment strategy, radiotherapy, poor histological features, incomplete bone resection, and tumor biopsy at the same institution as key prognostic factors.

Additionally, Alvarez-San Nicolas et al. [31] analyzed 90 cases of EWS in the limbs, discovering that poor treatment response, pelvic location, and the age group of 12–17 years were important prognostic factors. Wang et al. [32], in their analysis of 2059 cases of EWS and osteosarcoma, highlighted age, surgery, staging, primary location, tumor size, and histological type as critical factors influencing prognosis.

Zhan et al. [22] and Dai et al. [24] studied 1120 and 772 cases of EWS, respectively, finding that age, gender, primary location, tumor size, N stage, and M stage were significant prognostic factors. Zheng et al. [23], in their analysis of 1130 cases of EWS, found that younger patients, smaller tumors, absence of bone metastasis, and localized tumors had better prognoses, with surgery and chemotherapy positively impacting survival rates.

Other studies, such as those by Hsu et al., Chen et al., Jiang et al., Zhou et al., and Li et al. (2022) [23,25,33,34], also explored prognostic factors for EWS across different sample sizes and tumor locations, confirming the importance of factors such as age, race, tumor stage, surgery, chemotherapy, and metastasis.

For chordoma, Ouyang et al. [20] and Huang et al. [26] identified age, tumor size, histology, primary site, and the extent of surgical resection as critical determinants of prognosis in chordoma patients. Similarly, Li et al. [29] focused on chordomas of the pelvis and spine, underscoring the role of age, localized tumor involvement, and radical resection in influencing patient outcomes. Lin et al. [19] and Liu et al. [28] also reported that age, tumor size, disease extent, and surgical treatment were key prognostic factors, emphasizing the consistency of these findings across different studies and anatomical locations.

Moreover, studies by Teng et al. [35] and Huang et al. [27] explored chordomas located at the skull base and spine, respectively, and highlighted similar prognostic factors including primary site, disease stage, histological type, and the importance of surgical intervention. These studies collectively reinforce the significance of early diagnosis and comprehensive surgical management as cornerstones of effective chordoma treatment, regardless of tumor location.

Comparative studies between EWS and chordoma are limited, particularly those focusing on pelvic tumors. The SEER database, with its comprehensive collection of cancer incidence and survival data, provides an invaluable resource for examining the prognostic factors and survival outcomes of these rare malignancies. This study aims to fill the gap in the literature by providing a comparative analysis of pelvic EWS and chordoma, utilizing robust statistical methods to develop and validate predictive nomograms for OS, thereby aiding in the early identification of high-risk patients and informing clinical decision-making.

## 4 Methods

### 4.1 Data source and data extraction

In this study, we conducted a retrospective cohort study of patients diagnosed with pelvic EWS and chordoma. The SEER database is a population-based cancer registry that collects data on cancer incidence and survival from 17 cancer registries in the United States (2001–2019). Patient consent was waived for this observational study because it used de-identified data from the public SEER database.

**Fig. 1:**
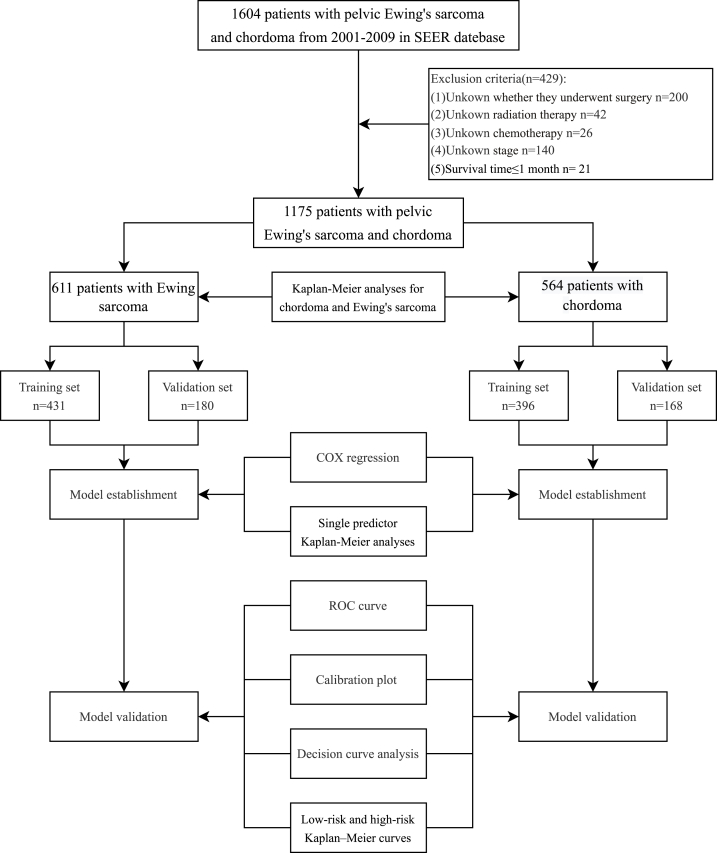
The flowchart of including and dividing patients.

Inclusion criteria were as follows: (1) Diagnosis confirmed by histopathological examination. (2) Ewing sarcoma and chordoma were diagnosed during the period from 2001 to 2019, with the pelvic bone as the site of origin. (3) Survival time is known and >1 month. The exclusion criteria were: (1) Unknown whether they underwent surgery. (2) Unknown radiation therapy. (3) Unknown chemotherapy. (4) Unknown stage.(5) Survival time≤1 month. A total of 1175 patients were eligible for final analysis. The inclusion and exclusion flowchart is shown in Fig. 1. Demographic and tumor data were obtained from the SEER*Stat software (version 8.4.0) for patients with pelvic EWS and chordoma.

We gathered patient demographic details, including age, gender, income, race, and regional origin; tumor characteristics, such as year of diagnosis, time from diagnosis to treatment, number of malignant tumors, and stage; treatment details, encompassing surgery, radiotherapy, and chemotherapy; and follow-up information, including survival status and survival time. Data extraction from electronic records of patients with pelvic EWS and chordoma at the SEER database was conducted by three trained researchers.

### 4.2 Outcome measures

The primary outcome of the survival analysis was overall survival (OS), defined as the time from diagnosis of pelvic EWS and chordoma to death from any cause. For individuals lost to follow-up before death, the last follow-up time is typically considered the time of death. OS was used to assess patients' survival status.

The survival curve, a Kaplan-Meier curve, depicts the survival status of patients over time. It is a crucial tool in survival analysis, enabling the comparison of survival differences between various cohorts.

Prognostic factors are characteristics associated with patient survival and can be used to predict the prognosis of individual patients.

### 4.3 Construction, validation of the nomogram and statistical analysis

Categorical variables were presented as frequencies and percentages (%) and compared using chi-squared tests. Univariate Cox regression analyses were performed to evaluate the association between each potential risk factor and overall survival (OS). Variables with a p-value <0.05 in the univariate analysis were included in the multivariate Cox regression analysis to identify independent predictors. Kaplan-Meier curves were generated to illustrate the impact of these independent risk factors on OS in patients with pelvic EWS and chordoma. Multicollinearity in the multivariate model was assessed using variance inflation factors (VIFs). Based on the multivariate analysis, a predictive nomogram for 3-, 5-, and 10-year OS for both EWS and chordoma was developed using R software in the training set.

To evaluate the nomogram's performance, a receiver operating characteristic (ROC) curve was plotted, and the area under the curve (AUC) was calculated to assess sensitivity and specificity. Calibration plots were generated to examine the nomogram's accuracy. Decision curve analysis (DCA) was used to assess the clinical utility of the predictive model by determining whether it improves forecasted net benefit. Patients were divided into low- and high-risk groups based on the nomogram score. Kaplan-Meier curves and log-rank analysis were used to compare survival differences between low-risk and high-risk groups.

Data analysis was conducted using SPSS version 26.0 (IBM Corp., USA) for statistical analysis and R version 4.0.3 (R Foundation for Statistical Computing, USA) for nomogram construction.

A graphical abstract has been included to visually summarize the main findings and contributions of our research (Supplementary eFig. 1).

## 5 Results

### 5.1 Baseline clinical and demographic characteristics of patients

A total of 1175 patients with pelvic EWS(n = 611) and chordoma(n = 564) were included. Pelvic EWS patients were randomly divided into training cohort 70 % (n = 431) and validation cohort 30 % (N = 180). Pelvic chordoma patients were also randomly divided into training cohort 70 % (n = 396) and validation cohort 30 % (n = 168)(Fig. 1).

Table 1 provides the baseline characteristics of the pelvic EWS and chordoma groups, respectively. Chordoma patients tended to be older than EWS patients (60 % vs 3 % aged ≥60 years). The gender ratio was similar between groups. There were no significant differences in income or region. Chordomas were more often diagnosed after 2008, while EWS predominated prior to 2008. Ewing's patients received faster treatment and had more metastatic disease at diagnosis, whereas chordomas were more localized. Ewing's patients received more chemotherapy and radiation; chordomas were more often managed with surgery. The baseline clinical and demographic characteristics of training cohort and validation cohort were similar, as shown in Table 2. Survival rates were significantly higher in the chordoma cohort compared to EWS group (3-year overall survival, 86.5 % VS 66.3 %, p < 0.001; 5-year survival was 79.4 % VS 59.4 %, p < 0.001; 10-year survival was 70.7 % SV 55.3 %, p < 0.001)(Table 3)

**Table 1:** Baseline clinical and demographic characteristics of the Pelvic Ewing's Sarcoma and Chordoma.

Variables	Total	Ewing's Sarcoma	Chordoma	p-value
	(1175)	(n = 611)	(n = 564)	
Age, years, n (%)				
<60	821 (70)	595 (97)	226 (40)	<0.001
≥60	354 (30)	16 (3)	338 (60)	
Sex, n (%)				
Male	456 (39)	366 (60)	353 (63)	0.377
Female	719 (61)	245 (40)	211 (37)	
Income (dollar), n (%)				
≥75000	173 (15)	95 (16)	78 (14)	0.503
55000-75000	498 (42)	263 (43)	235 (42)	
<55000	504 (43)	253 (41)	251 (45)	
Region, n (%)				
≥1 million pop area	787 (67)	406 (66)	381 (68)	0.331
<1 million pop area	288 (25)	146 (24)	142 (25)	
Nonmetropolitan	100 (9)	59 (10)	41 (7)	
Race, n (%)				
White	1005 (86)	531 (87)	474 (84)	0.263
Black	50 (4)	21 (3)	29 (5)	
Other	120 (10)	59 (10)	61 (11)	
Year of diagnosis, n (%)				
2001–2008	238 (20)	148 (24)	90 (16)	<0.001
2008–2014	424 (36)	221 (36)	203 (36)	
2015–2019	513 (44)	242 (40)	271 (48)	
Months from diagnosis to treatment, n (%)				
0	342 (29)	255 (42)	87 (15)	<0.001
1	285 (24)	191 (31)	94 (17)	
2	235 (20)	37 (6)	198 (35)	
≥3	313 (27)	128 (21)	185 (33)	
The number of malignant tumor, n (%)				
1	1020 (87)	584 (96)	436 (77)	<0.001
2	122 (10)	24 (4)	98 (17)	
≥3	33 (3)	3 (0)	30 (5)	
Stage, n (%)				
Localized	354 (30)	105 (17)	249 (44)	<0.001
Regional	459 (39)	198 (32)	261 (46)	
Distant	362 (31)	308 (50)	54 (10)	
Radiation, n (%)				
No	606 (52)	256 (42)	350 (62)	<0.001
Yes	569 (48)	355 (58)	214 (38)	
Chemotherapy, n (%)				
No	561 (48)	24 (4)	537 (95)	<0.001
Yes	614 (52)	587 (96)	27 (5)	
Surgery, n (%)				
No	535 (46)	160 (26)	375 (66)	<0.001
Yes	640 (54)	451 (74)	189 (34)	

p-value is from Chi-Squared Test to indicate significant differentiation (P < 0.05 means significant differentiation).

**Table 2:** Baseline clinical and demographic characteristics of training and validation set in the pelvic Ewing's Sarcoma and Chordoma.

Variables	Ewing's Sarcoma (n = 611)			Chordoma (n = 564)		
	Training set	Validation set	p	Training set	Validation	p
	(n = 431)	(n = 180)		(n = 396)	set (n = 168)	
Age, years, n (%)						
<60	421 (98)	174 (97)	0.795	159 (40)	67 (40)	0.994
≥60	10 (2)	6 (3)		237 (60)	101 (60)	
Sex, n (%)						
Male	249 (58)	117 (65)	0.114	252 (64)	101 (60)	0.294
Female	182 (42)	63 (35)		144 (36)	67 (40)	
Income (dollar), n (%)						
≥75000	69 (16)	26 (14)	0.338	55 (14)	23 (14)	0.331
55000-75000	193 (45)	70 (39)		165 (42)	70 (42)	
<55000	169 (39)	84 (47)		176 (44)	75 (45)	
Region, n (%)						
≥1 million pop area	289 (67)	117 (65)	0.357	262 (66)	119 (71)	0.118
<1 million pop area	106 (25)	40 (22)		103 (26)	39 (23)	
Nonmetropolitan	36 (8)	23 (13)		31 (8)	10 (6)	
Race, n (%)						
White	374 (87)	157 (87)	1.000	333 (84)	141 (84)	0.984
Black	15 (3)	6 (3)		25 (6)	4 (2)	
Other	42 (10)	17 (10)		38 (10)	23 (14)	
Year of diagnosis, n (%)						
2001–2008	97 (23)	51 (28)	0.329	64 (16)	26 (15)	0.119
2008–2014	161 (37)	60 (33)		146 (37)	57 (34)	
2015–2019	173 (40)	69 (38)		186 (47)	85 (51)	
Months from diagnosis to treatment, n (%)						
0	175 (41)	80 (44)	0.983	59 (15)	28 (17)	0.728
1	139 (32)	52 (29)		68 (17)	26 (15)	
2	27 (6)	10 (6)		142 (36)	56 (33)	
≥3	90 (21)	38 (21)		127 (32)	58 (35)	
The number of malignant tumor, n (%)						
1	411 (95)	173 (96)	0.537	306 (77)	130 (77)	0.687
2	18 (4)	6 (3)		66 (17)	32 (19)	
≥3	2 (0)	1 (1)		24 (6)	6 (4)	
Stage, n (%)						
Localized	80 (19)	25 (14)	0.444	179 (45)	70 (42)	0.250
Regional	134 (31)	64 (36)		178 (45)	83 (49)	
Distant	217 (50)	91 (51)		1.70(0.93)	15 (9)	
Radiation, n (%)						
No	182 (42)	74 (41)	0.845	156(49.2)	105 (62)	0.915
Yes	249 (58)	106 (59)		161(50.8)	63 (38)	
Chemotherapy, n (%)						
No	15 (3)	9 (5)	0.360	3.94(0.66)	163 (97)	0.338
Yes	416 (97)	171 (95)		8.61(2.80)	5 (3)	
Surgery, n (%)						
No	116 (27)	44 (24)	0.619	1.30(0.55)	112 (67)	0.800
Yes	315 (73)	136 (76)		201.97(72.63)	56 (33)	

p < 0.05: statistically significant difference.

**Table 3:** Comparison of the mortality rate of the pelvic Ewing's Sarcoma and Chordoma.

Status	3-year		P^a^-value	5-year		P^a^-value	10-year		P^a^-value
	Ewing's Sarcoma	Chordoma		Ewing's Sarcoma	Chordoma		Ewing's Sarcoma	Chordoma	
	(n = 611)	(n = 564)		(n = 611)	(n = 564)		(n = 611)	(n = 564)	
Alive	405(66.3)	488(86.5)	<0.001	363(59.4)	488(79.4)	<0.001	338(55.3)	399(70.7)	<0.001
Dead	206(33.7)	76(13.5)		248(40.6)	116(20.6)		273(44.7)	165(29.3)	

a p-value is from Chi-Squared Test to indicate significant differentiation (P < 0.05 means significant differentiation).

### 5.2 Independent risk factors in the training set

**Fig. 2:**
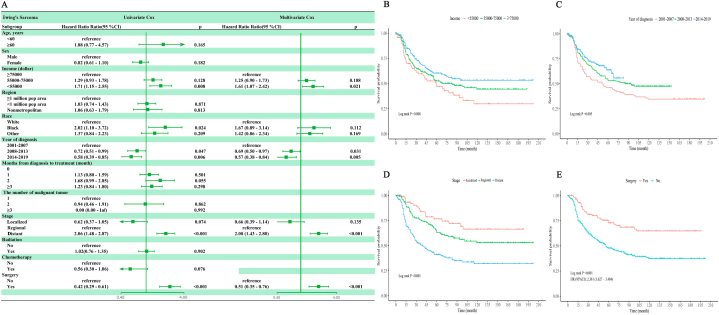
(A)Univariate and multivariate analyses of Pelvic Ewing's Sarcoma. (B) Kaplan–Meier curves of Pelvic EWS in different income groups. (C) Kaplan–Meier curves of Pelvic EWS in different years of diagnosis groups. (D) Kaplan–Meier curves of Pelvic EWS in different stage groups. (E) Kaplan–Meier curves of Pelvic EWS in with or without surgery groups.

**Fig. 3:**
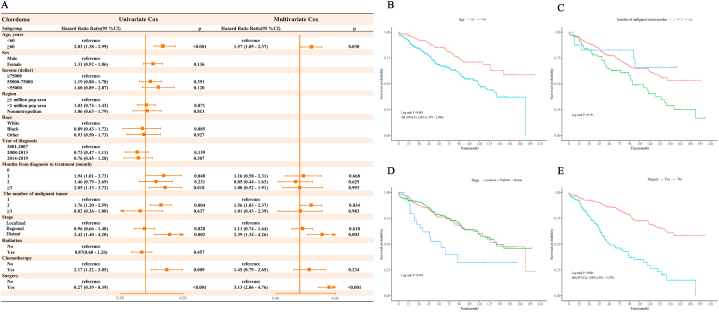
(A)Univariate and multivariate analyses of Chordoma. (B) Kaplan–Meier curves of Pelvic Chordoma in different age groups. (C) Kaplan–Meier curves of Pelvic Chordoma in different numbers of malignant tumor groups. (D) Kaplan–Meier curves of Pelvic Chordoma in different stage groups. (E) Kaplan–Meier curves of Pelvic Chordoma in with or without surgery groups.

Univariate and multivariate COX regression analyses revealed several independent risk factors for OS in patients with pelvic EWS and chordoma, respectively. For pelvic EWS, these factors included demonstrated year of diagnosis, income, stage and surgery were significantly associated with survival (Fig. 2A). For chordoma, age, months from diagnosis to treatment, stage and surgery were significantly associated with survival (Fig. 3A). Kaplan-Meier curves depict the influence of these independent risk factors on OS in patients with pelvic EWS and chordoma (Fig. 2, Fig. 3B–E). The variance inflation factors (VIFs) for these risk factors ranged from 1.07 to 1.62 for EWS and from 1.05 to 1.49 for chordoma, indicating the absence of multicollinearity.

### 5.3 Nomogram model establishment

**Fig. 4:**
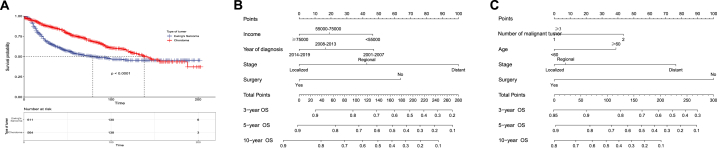
(A) Kaplan–Meier curves of Pelvic EWS and Chordoma groups. (B) Nomogram for 3-, 5-, and 8-year OS of Pelvic EWS. (C) Nomogram for 3-, 5-, and 8-year OS of Pelvic Chordoma.

Kaplan-Meier curves depict the influence of pelvic EWS and chordoma on OS in patients with pelvic EWS and chordoma (Fig. 4A). Drawing upon multivariate Cox regression analysis, we constructed nomogram models to predict overall survival (OS) for pelvic EWS and chordoma. The EWS nomogram comprises four independent risk factors: year of diagnosis, income, stage, and surgery. The chordoma nomogram also includes four independent risk factors: age, time from diagnosis to treatment, stage, and surgery. Each nomogram factor is assigned a corresponding score, with the overall score reflecting OS. Stage had the greatest impact on EWS prognosis, while surgery had the most significant influence on chordoma prognosis (Fig. 4B and C).

### 5.4 Nomogram model validation

**Fig. 5:**
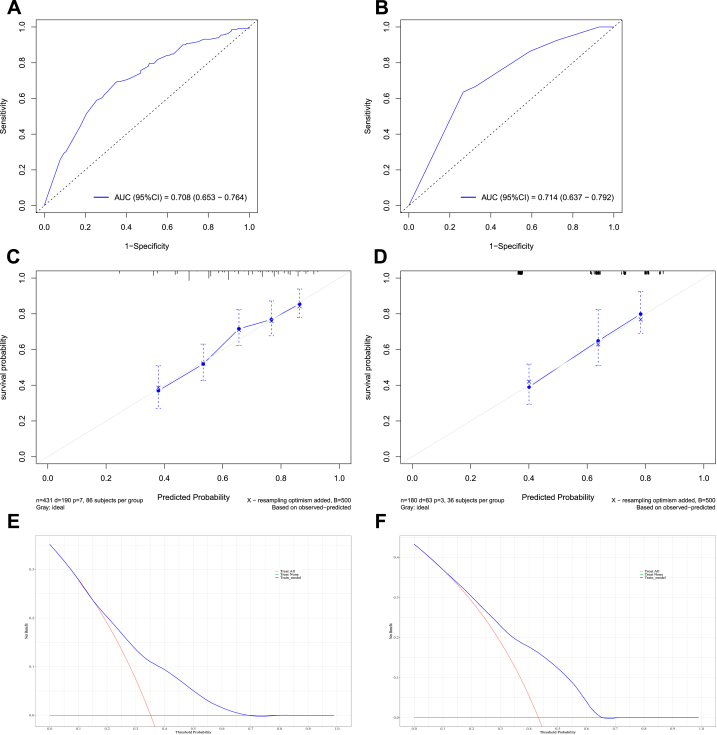
The ROC, calibration curve and decision curves of the nomogram predicting 3-year OS of Pelvic EWS in the training set (A, C and D) and the validation set (B, D and F).

**Fig. 6:**
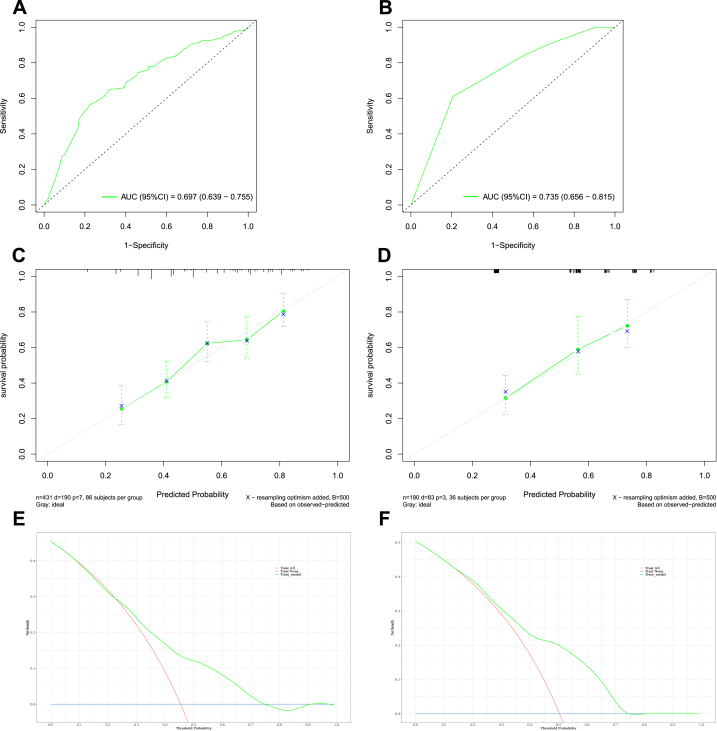
The ROC, calibration curve and decision curves of the nomogram predicting 5-year OS of Pelvic EWS in the training set (A, C and D) and the validation set (B, D and F).

**Fig. 7:**
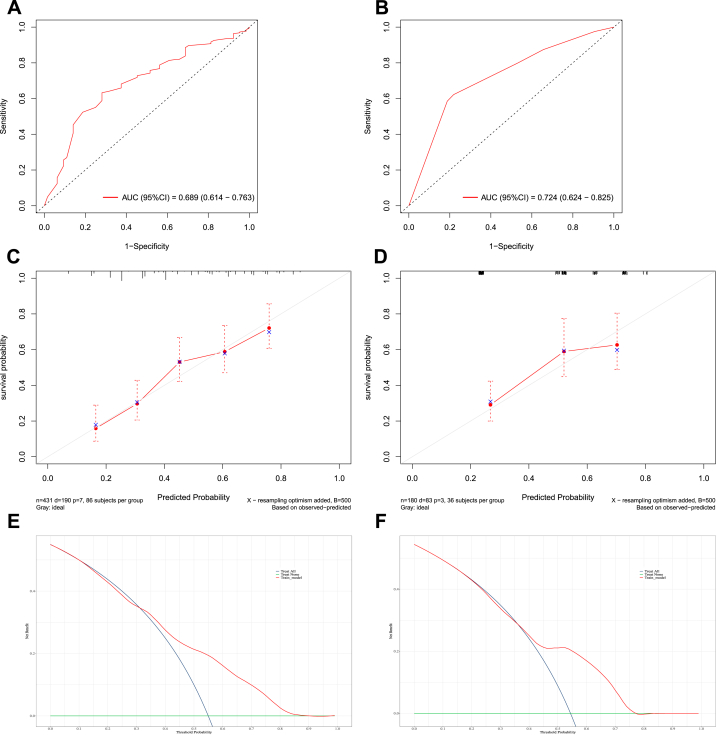
The ROC, calibration curve and decision curves of the nomogram predicting 10-year OS of Pelvic EWS in the training set (A, C and D) and the validation set (B, D and F).

**Fig. 8:**
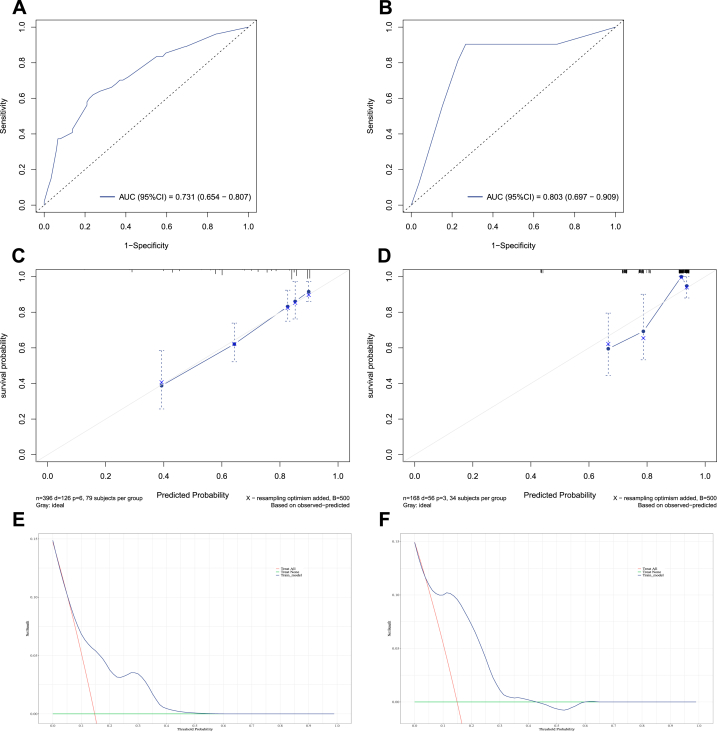
The ROC, calibration curve and decision curves of the nomogram predicting 3-year OS of Pelvic Chordoma in the training set (A, C and D) and the validation set (B, D and F).

**Fig. 9:**
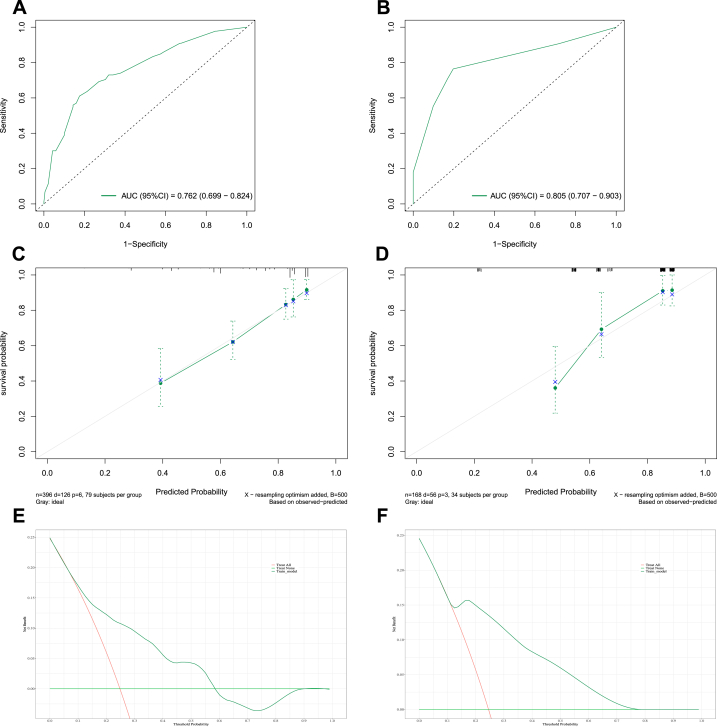
The ROC, calibration curve and decision curves of the nomogram predicting 5-year OS of Pelvic Chordoma in the training set (A, C and D) and the validation set (B, D and F).

**Fig. 10:**
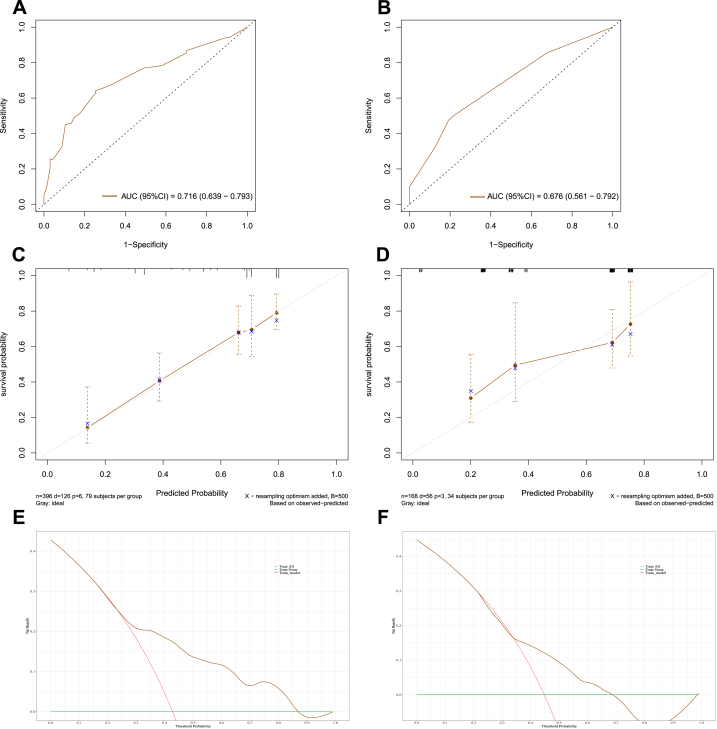
The ROC, calibration curve and decision curves of the nomogram predicting 10-year OS of Pelvic Chordoma in the training set (A, C and D) and the validation set (B, D and F).

In the training set, the area under the curve (AUC) values reflecting overall survival prediction accuracy were 0.708, 0.697, and 0.689 for 3-, 5-, and 10-year survival in EWS (Fig. 5, Fig. 6, Fig. 7A). In the validation sets, the AUC values were 0.717, 0.735, and 0.724, respectively(Fig. 5, Fig. 6, Fig. 7B). Similarly, the AUC values in the training set were 0.731, 0.762, and 0.716 for 3-, 5-, and 10-year overall survival in chordoma patients (Fig. 8, Fig. 9, Fig. 10A). The corresponding AUC values in the validation sets were 0.803, 0.805, and 0.676 (Fig. 8, Fig. 9, Fig. 10B).

Calibration plots demonstrated strong agreement between nomogram-predicted and observed outcomes for 3-, 5-, and 10-year survival in both the training and validation sets (C for training sets and D for validation sets**)** for Ewing sarcoma (Fig. 5, Fig. 6, Fig. 7C&D) and chordoma (Fig. 8, Fig. 9, Fig. 10C&D). Similarly, decision curve analysis showed that the Ewing sarcoma and chordoma nomograms conferred greater net benefit over non-intervention across a range of threshold probabilities for 3-, 5-, and 10-year survival in the training and validation sets (Ewing sarcoma Fig. 5, Fig. 6, Fig. 7E&F; chordoma Fig. 8, Fig. 9, Fig. 10E&F) (E for training sets and F for validation sets).

**Fig. 11:**
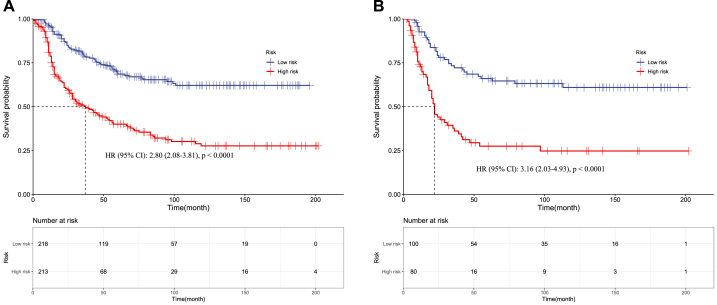
Kaplan–Meier curves of Pelvic EWS in the low- and high-risk groups in the training set **(A)** and validation set **(B).**

**Fig. 12:**
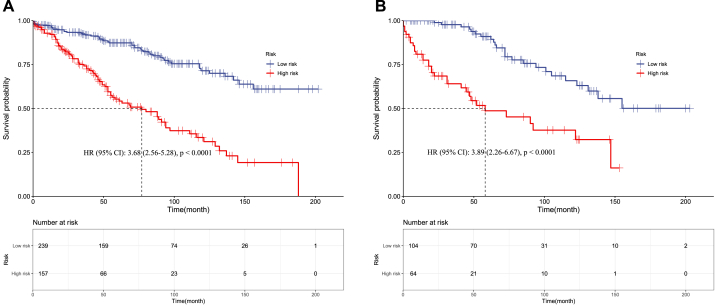
Kaplan–Meier curves of Pelvic Chordoma in the low- and high-risk groups in the training set **(A)** and validation set **(B)**.

In EWS, the high-risk group has significantly worse survival compared to the low-risk group in both the training set (HR 2·80, 95 % CI 2·08–3·81; p < 0·0001) (Fig. 11A) and validation set (HR 3·16, 2·03–4·93; p < 0·0001) (Fig. 11B). The median survival is markedly reduced for the high-risk versus low-risk EWS patients. Similarly, chordoma high-risk patients had significantly worse survival than low-risk patients in the training (HR 3·68, 2·56–5·28; p < 0·0001) (Fig. 12A) and validation sets (HR 3·89, 2·26–6·67; p < 0·0001) (Fig. 12B).

These metrics substantiate the accurate prediction of 3-, 5-, and 10-year overall survival (OS) for both Ewing sarcoma and chordoma using the respective nomograms. The two nomograms showed strong discrimination and prediction performance, supporting their reliability as valuable clinical tools.

## 6 Discussion

Our study using the SEER database demonstrates key differences in prognostic factors and overall survival between pelvic EWS and chordoma. Multivariate analysis revealed distinct factors independently influencing overall survival for each malignancy.

For EWS, the year of diagnosis, income status, tumor stage, and surgical treatment were significant in prognosis. The shift towards improved survival over time likely reflects advancement in multimodal therapy incorporating chemotherapy and radiation. Studies have shown that multimodal therapy consisting of radiation therapy to all areas of gross disease and intensive combination chemotherapy has led to complete responses and improved disease-free outcomes in patients with EWS [[36], [37], [38]]. For instance, in a study of nonpelvic, localized EWS of bone, the addition of Adriamycin or bilateral pulmonary radiotherapy to vincristine, dactinomycin, and cyclophosphamide therapy significantly improved survival rates [36]. Similarly, in the management of localized EWS of pelvic and sacral bones, multimodal therapy resulted in a significant advantage in relapse-free survival and overall survival compared to historical control series [37].

Interestingly, higher income was associated with better outcomes in EWS, suggesting potential disparities in access to optimal cancer care. Alsoof et al. observed income was significantly associated with decreased survival in patients with Ewing sarcoma in a prospective observational study (p < 0.001) [39]. Panda et al. conducted a prospective study of 66 patients with Ewing sarcoma in adults over 40 years of age from a low-middle-income country and found 5-year OS were 48.1 % (95 % CI = 34.6–61.6) [40].

Those with higher incomes tend to have better access to high-quality healthcare. They can more easily afford comprehensive health insurance that covers advanced treatments [40,41]. This includes access to major cancer centers and clinical trials for emerging therapies. The financial resources also allow them to pay for expenses not fully covered, like travel costs for care. In addition, those with more disposable income can likely maintain healthier lifestyles. A nutritious diet, regular exercise, and lower stress levels may improve resilience during intensive cancer treatments. Avoiding delays in seeking care due to costs can also lead to earlier diagnosis at more treatable stages.

As expected, lower tumor stage and surgical resection conferred survival benefits. Our research findings are consistent with many previous studies. Compared to existing literature, this study provides a more comprehensive perspective.

Additionally, Fizazi et al. found that metastasis at presentation (P = 0.00001) was an independent prognostic factor for survival in a retrospective cohort study of 182 patients [42]. Joe Lee et al. indicated metastatic disease remained a significant prognostic factor in Ewing sarcoma(HR, 2.74; 95 % CI, 2.14–3.49). The study conducted by Verma found that metastatic disease is associated with OS [43].

In contrast, for chordomas, we analyzed prognostic factors for pelvic chordoma and identified age, time from diagnosis to treatment, tumor stage, and surgical intervention as significant predictors of overall survival in this study. These findings align with the broader literature on chordoma, which consistently highlights the importance of these factors.

Additionally, chordomas characteristically affect older adults, whose comorbidities and performance status may hinder intensive treatment [44]. Expedient management is prudent to avoid further local spread [45]. Similarly, achieving surgical resection is a consistent prognostic factor, but challenging in the pelvis [46].

Beyond differences in prognostic variables, overall survival was significantly higher across all timepoints for chordomas compared to EWS of the pelvis. This survival advantage may relate to chordomas' lack of metastatic potential, allowing primarily local control to impact outcomes [45]. Conversely, metastatic disease burdens prognosis in a substantial proportion of Ewing's patients [47,48].By delineating distinct prognostic profiles, our nomograms provide practical clinical tools to risk stratify pelvic Ewing's and chordoma patients. Accurate assessment of expected outcomes facilitates personalized treatment decisions incorporating patient goals. For example, a young patient with extensive metastatic Ewing may elect palliative radiation over intensive chemotherapy in light of poor predicted survival. An older chordoma patient with medical issues could pursue a course of definitive radiation therapy rather than a high-risk resection.

## 7 Limitations and strengths

Several limitations should be acknowledged regarding the SEER database and retrospective analyses. This study has several limitations that should be acknowledged. Firstly, the retrospective nature of the analysis using the SEER database inherently carries the potential for selection bias and missing data, which may influence the findings. Secondly, the SEER database lacks detailed information on certain variables that could impact outcomes, such as specific chemotherapy regimens, radiation doses, surgical margins, and molecular characteristics of the tumors. Thirdly, the database does not provide comprehensive data on recurrence or progression-free survival, which are important endpoints in cancer research. Additionally, while the SEER database includes a diverse population, it may not fully represent the entire U.S. population or capture the nuances of care in different healthcare settings. The follow-up period for some patients may not be sufficient to observe long-term outcomes, particularly for chordoma, which is known for its slow progression and late recurrences. Lastly, we did not conduct a more detailed subgroup analysis, such as age.

This study has several strengths. Firstly, it utilizes the large and diverse SEER database, enhancing the generalizability of the findings. The data from 2001 to 2019 provide a comprehensive view of long-term trends and outcomes. Secondly, strict inclusion criteria ensure accurate analysis of well-defined, histopathologically confirmed cases. Multivariate Cox regression identifies key prognostic factors, offering deeper insights into survival influences. Additionally, the validated nomogram for 3-, 5-, and 10-year overall survival provides a useful tool for personalized patient management. The comparative analysis of pelvic EWS and chordoma offers valuable insights for better treatment strategies. Finally, rigorous statistical methods, including Kaplan-Meier and ROC curves, ensure a thorough and credible evaluation of survival outcomes.

### 7.1 Future works

Future research should focus on several key areas. Prospective studies with larger sample sizes are needed to validate our findings and explore additional survival factors. Detailed molecular and genetic analyses could identify new biomarkers and treatment targets, leading to more personalized therapies. Investigating the impact of specific treatments, including chemotherapy types, advanced radiation techniques, and surgical innovations, could optimize therapy for these rare tumors. Long-term follow-up studies are essential to understand the natural history and durability of treatment responses, especially for chordoma.

In summary, our study delineates distinct prognostic variables in pelvic EWS versus chordomas. Developed nomograms provide means to predict overall survival based on these factors, with the potential to guide individualized treatment decisions. Findings highlight the differing biology and clinical behavior of these rare malignancies.

## 8 Conclusion

This study successfully developed and validated nomograms for predicting overall survival (OS) in patients with pelvic EWS and chordoma, identifying key prognostic factors and comparing survival outcomes between these two rare conditions. The higher survival rates observed in chordoma patients compared to those with EWS emphasize the need for different strategies in treating these tumors.

## Ethics approval and consent to participate

For this type of study formal consent is not required and the Institutional Review Board of Dandong Central Hospital waived the need for informed consent. All procedures performed in studies involving human participants were in accordance with the ethical standards of the Institutional Review Board of Dandong Central Hospital and with the 1964 Helsinki declaration and its later amendments or comparable ethical standards.

## Consent for publication

Not applicable.

## Data availability statement

Publicly available datasets were analyzed in this study. This data can be found here: https://seer.Cancer.gov/.

## Funding

None.

## CRediT authorship contribution statement

**Wanyun Tang:** Funding acquisition, Formal analysis, Conceptualization. **Runzhuo Li:** Data curation. **Xiaoying Lai:** Conceptualization. **Xiaohan Yu:** Data curation. **Renjian He:** Conceptualization.

## Declaration of competing interest

The authors declare that they have no known competing financial interests or personal relationships that could have appeared to influence the work reported in this paper.
