# Supplementary figures and images for: Prognostic factors and overall survival in pelvic Ewing's sarcoma and chordoma: A comparative SEER database analysis
Source: Heliyon. 2024 Aug 28;10(17):e37013. doi: 10.1016/j.heliyon.2024.e37013 (PMC11402751; doi:10.1016/j.heliyon.2024.e37013)

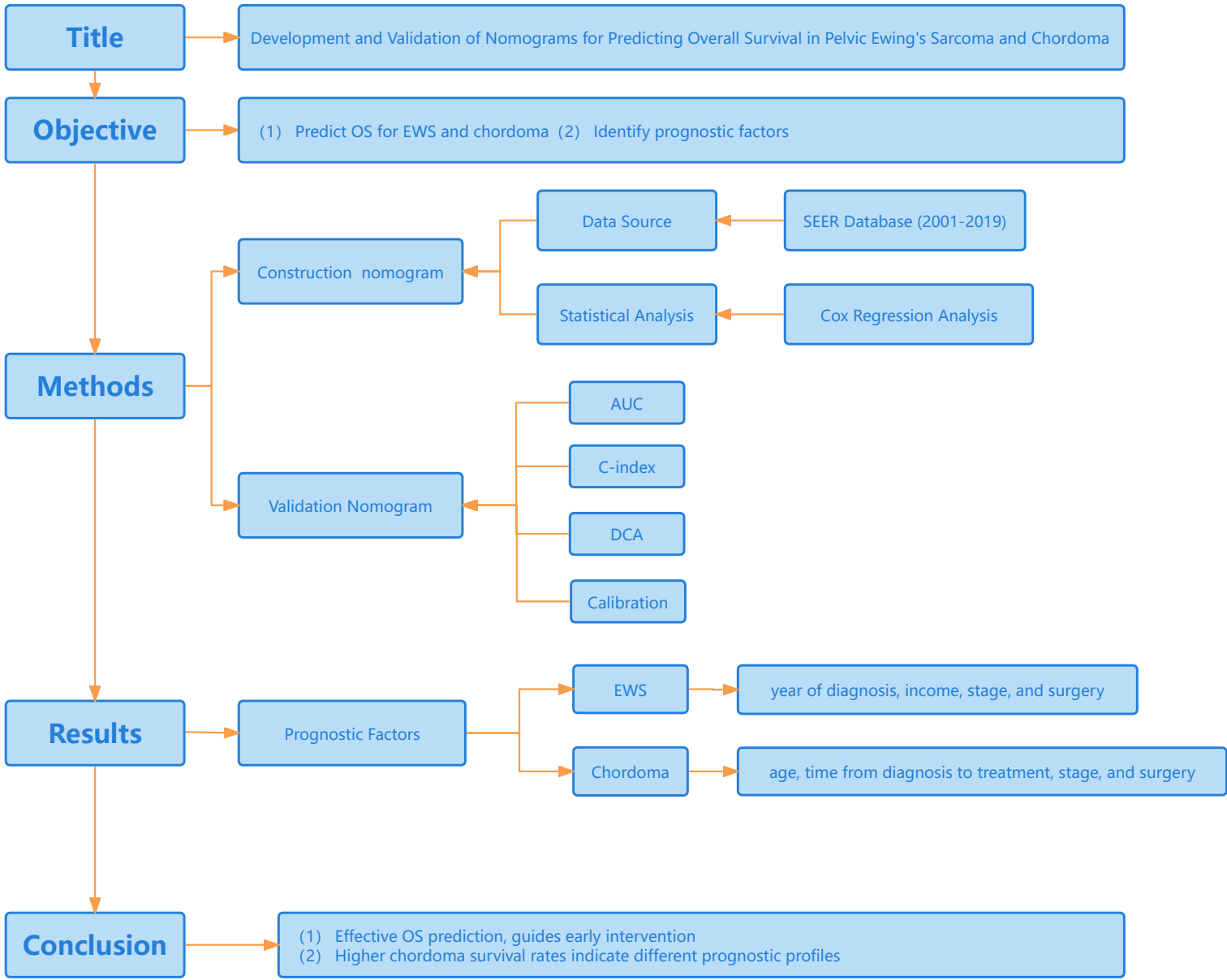

Supplement: Multimedia component 1 [file mmc1.pdf]
