# Supplementary material for: Prognostic factors and overall survival in pelvic Ewing's sarcoma and chordoma: A comparative SEER database analysis
Source: Heliyon. 2024 Aug 28;10(17):e37013. doi: 10.1016/j.heliyon.2024.e37013 (PMC11402751; doi:10.1016/j.heliyon.2024.e37013)
Supplement: Multimedia component 2 [file mmc2.docx]

**Supplementary Table 1 Summarizes the key features of our current study in comparison with existing studies.**

| Study | Study type | Sample size | Location of tumor occurrence | Tumor type | Prognostic factors | Type of validation |
| --- | --- | --- | --- | --- | --- | --- |
| Our study | Retrospective | 1175 | Pelvic | Ewing's Sarcoma and Chordoma | For pelvic EWS: demonstrated year of diagnosis, income, stage and surgery  For chordoma: age, months from diagnosis to treatment, stage and surgery | internal validation |
| Mathew et al. (2022)  10.1245/s10434-022-12992-1 | Retrospective | 47 | Extraskeletal | Ewing's Sarcoma | trunkal location, margin-positive resections | - |
| [Dimosthenis Andreou](https://pubmed.ncbi.nlm.nih.gov/?term=Andreou+D&cauthor_id=31580267) et al. (2020)  DOI:10.1245/s10434-022-12992-1 | Retrospective | 1411 | Pelvic | Ewing's Sarcoma | Tumor site, Treatment strategies， Radiotherapy, Poor Histologic,  Incomplete Bone Resection, Tumor Biopsy at Same Institution as Resection | - |
| Alvarez-SanNicolas et al. ( 2019 )  10.1007/s12094-019-02067-1 | Retrospective | 90 | limb | Ewing's Sarcoma | poor response to treatment, pelvis location and age between 12 and 17 years | - |
| [Wang](https://pubmed.ncbi.nlm.nih.gov/?term=Wang+J&cauthor_id=35178367) et al.（2022）  10.3389/fpubh.2022.837506 | Retrospective | 2059 | - | Ewing's Sarcoma and Osteosarcoma | age, surgery, stage, primary site, tumor size, and histological type | internal validation |
| Zhan et al.（2021）  10.1038/s41598-021-02134-0 | Retrospective | 1120 | - | Ewing’s sarcoma | age, gender, primary site, tumor size, N stage, and M stage | internal validation |
| Zheng et al.（2022）  10.1038/s41598-022-11827-z | Retrospective | 1130 | - | Ewing’s sarcoma | young patients (≤ 18 years old), small tumour size (≤ 58 mm), no/unknown bone metastasis, localized tumour stage, and received surgery and chemotherapy | internal validation |
| Dai et al.（2022）  10.2147/IJGM.S324163 | Retrospective | 772 | - | Ewing’s sarcoma | Age, race, tumor size, and tumor stage | internal validation |
| Hsu et al.（2023）  10.3389/fped.2023.1103565 | Retrospective | 756 | - | Ewing’s sarcoma | age, T stage, N stage, M stage, surgery, and chemotherapy | - |
| Chen et al.（2019）  10.7717/peerj.7710 | Retrospective | 267 | Pelvic | Ewing’s sarcoma | Age, race, tumor stage, and surgery | - |
| Jiang et al.（2021）  10.1097/BRS.0000000000004022 | Retrospective | 371 | Pelvis and Spine | Ewing’s sarcoma | age, tumor extent, tumor size, and surgical treatments | - |
| Zhou  et al.（2020）  10.1186/s12891-020-03706-3 | Retrospective | 578 | - | Ewing’s sarcoma | age, N stage and bone metastasis | internal validation |
| Li  et al.（2022）  10.1186/s12885-022-09796-7 | Retrospective | 767 | - | Ewing’s sarcoma | age, bone metastasis, tumor size, and chemotherapy | Internal and external validation |
| Ouyang et al.（2024）  10.1177/10225536241254208 | Retrospective | 658 | - | chordoma | age, tumor size, histology, primary site, surgery, and extent of disease | Internal validation |
| Li et al.（2023）  10.1007/s00586-023-07590-y | Retrospective | 485 | Pelvis and Spine | chordoma | age, localized involvement, and radical resection | Internal validation |
| Lin et al.（2020）  10.1016/j.clineuro.2020.106174 | Retrospective | 294 | Spine | chordoma | age at diagnosis, tumor size, extent of disease, and treatment | Internal validation |
| Huang et al.（2019）  10.1016/j.wneu.2019.04.217 | Retrospective | 425 | - | chordoma | age, sex, race, disease stage, surgery, year of diagnosis, marital status, primary site, radiation, and tumor size | - |
| Teng et al.（2021）  10.3389/fsurg.2021.764329 | Retrospective | 643 | Skull Base | chordoma | age at diagnosis, primary site, disease stage, surgical treatment, and tumor size | external validation |
| Liu et al.（2022）  10.2217/fon-2022-0158 | Retrospective | 362 | - | chordoma | disease stage, age, surgery, marital status and tumor size | - |
| Huang et al.（2021）  10.1177/15330338211036533 | Retrospective | 316 | Spine | chordoma | Primary site, disease stage, histological type, surgery, and age | Internal validation |
